# Supplementary material for: Novel Driver Strength Index highlights important cancer genes in TCGA PanCanAtlas patients
Source: PeerJ. 2022 Aug 11;10:e13860. doi: 10.7717/peerj.13860 (PMC9375969; doi:10.7717/peerj.13860)
Supplement: Supplemental Information 2 [file peerj-10-13860-s002.zip › COHORTS/genes plots/detailed/2021_8_16_14_9_distribution_events_detailed_males_40.pdf]

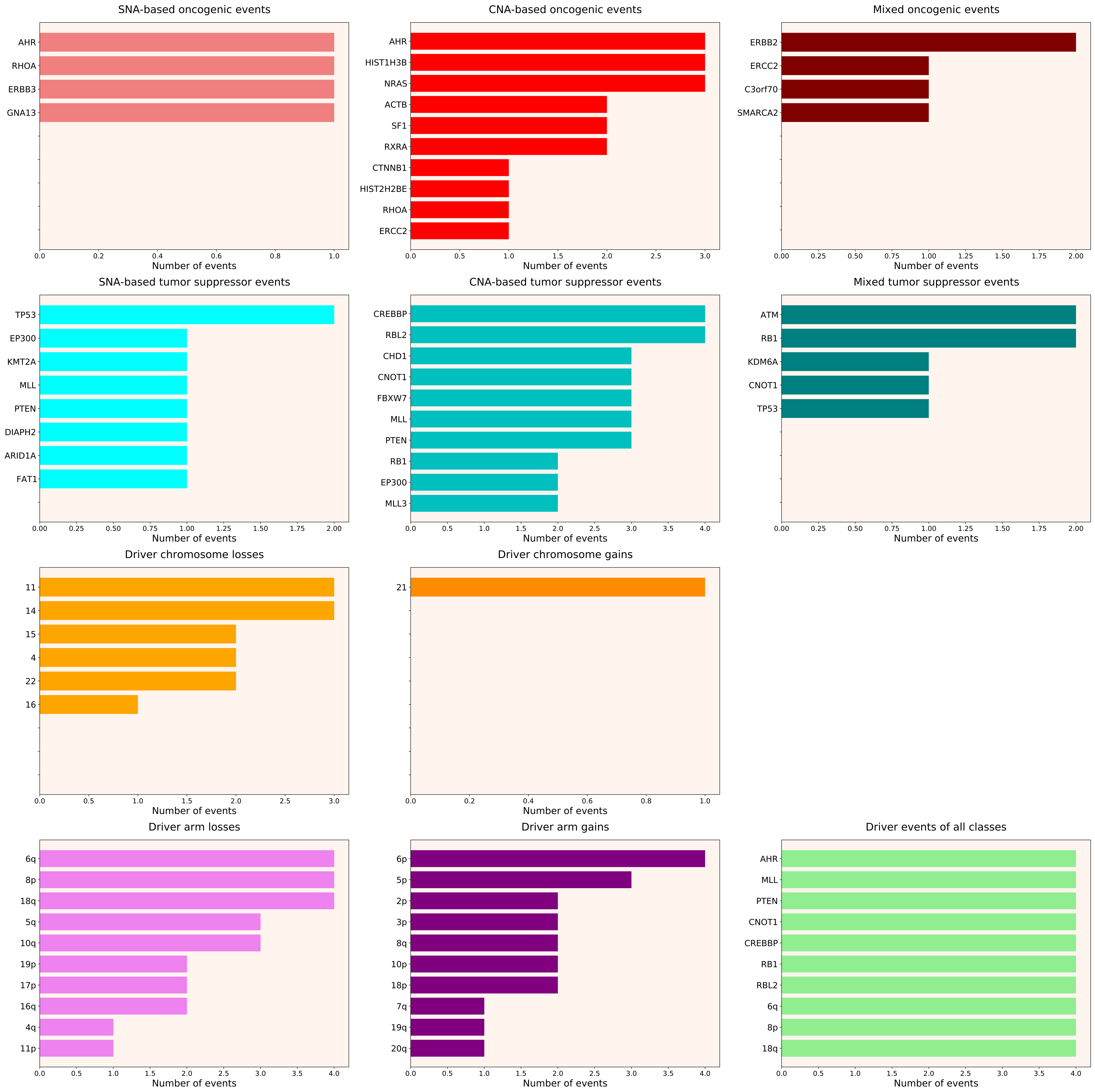

SNA-based oncogenic events
AHR
RHOA
ERBB3
GNA13
Number of events
CNA-based oncogenic events
AHR
HIST1H3B
NRAS
ACTB
SF1
RXRA
CTNNB1
HIST2H2BE
RHOA
ERCC2
Number of events
Mixed oncogenic events
ERBB2
ERCC2
C3orf70
SMARCA2
Number of events
SNA-based tumor suppressor events
TP53
EP300
KMT2A
MLL
PTEN
DIAPH2
ARID1A
FAT1
Number of events
CNA-based tumor suppressor events
CREBBP
RBL2
CHD1
CNOT1
FBXW7
MLL
PTEN
RB1
EP300
MLL3
Number of events
Mixed tumor suppressor events
ATM
RB1
KDM6A
CNOT1
TP53
Number of events
Driver chromosome losses
11
14
15
4
22
16
Number of events
Driver chromosome gains
21
Number of events
Driver arm losses
6q
8p
18q
5q
10q
19p
17p
16q
4q
11p
Number of events
Driver arm gains
6p
5p
2p
3p
8q
10p
18p
7q
19q
20q
Number of events
Driver events of all classes
AHR
MLL
PTEN
CNOT1
CREBBP
RB1
RBL2
6q
8p
18q
Number of events
